# Spatial dependence of non-traumatic out-of-hospital cardiac arrest in a Swiss region: A retrospective analysis

**DOI:** 10.1016/j.resplu.2024.100713

**Published:** 2024-07-13

**Authors:** Guillaume Lengen, Olivier Hugli, David De Ridder, Idris Guessous, Anaïs Ladoy, Stéphane Joost, Pierre-Nicolas Carron

**Affiliations:** a University of Lausanne, Faculty of Biology and Medicine, 21 Rue du Bugnon, 1005 Lausanne, Switzerland; b Emergency Department, Lausanne University Hospital and University of Lausanne, 46 Rue du Bugnon, 1011 Lausanne, Switzerland; c Geospatial Molecular Epidemiology Group, Laboratory for Biological Geochemistry, School of Architecture, Civil and Environmental Engineering, Ecole Polytechnique Fédérale de Lausanne, 1015 Lausanne, Switzerland; d Group of Geographic Information Research and Analysis in Population Health, Switzerland; e Unit of Population Epidemiology, Division and Department of Primary Care Medicine, Department of Community Medicine, Primary Care and Emergency Medicine, Geneva University Hospitals, 4 Rue Gabrielle-Perret-Gentil, 1211 Geneva 14, Switzerland; f ‘La Source’ School of Nursing, University of Applied Sciences and Arts Western Switzerland, Lausanne, Switzerland

**Keywords:** OHCA, BCPR, Geographic Information Systems, Spatial dependence, Incidence, Survival

## Abstract

**Background:**

Out-of-hospital cardiac arrest (OHCA) incidence and survival often vary within regions according to patient-related and contextual factors. This study aims to establish the overall spatial dependence of incidence, bystander cardiopulmonary resuscitation (BCPR) and 48-h survival of OHCA with their associated demographic and socioeconomic characteristics in a Swiss region.

**Methods:**

We conducted a retrospective study using data of all OHCAs recorded between 2007 and 2019 in the canton of Vaud and, more specifically, in the Lausanne area. Provision of BCPR and 48-h survival were analysed using Getis-Ord Gi statistics and OHCA incidence by local Moran’s I with empirical Bayes standardised rates. Demographic and socioeconomic characteristics were compared between incidence clusters generated by local Moran’s I method.

**Results:**

Significant spatial variations of OHCA incidence, BCPR and 48-h mortality were observed. Although BCPR was statistically more likely in rural areas, 48-h survival was improved in a few main cities. At the cantonal level, postcode areas with a higher incidence of OHCAs were less densely inhabited with lower salary levels, more Swiss citizens, and an older population. At city level, small area variations were detected within urban neighbourhoods. The more affected hectares with more OHCAs were less inhabited, with a better median salary, more Swiss citizens, and off-centre.

**Conclusions:**

Spatial variations associated with demographic and socioeconomic factors were observed for OHCA incidence and survival, with sparsely populated areas particularly at risk. These data suggest an unmet need for targeted prevention interventions and structural modifications of the existing prehospital system at the cantonal level.

## Highlights

• Significant spatial variations of out-of-hospital cardiac arrest incidence, bystander cardiopulmonary resuscitation and 48-h mortality were observed in a Swiss region.
• High risks areas of out-of-hospital cardiac arrest tend to be outside the city centre and are correlated with socioeconomic and sociodemographic characteristics.
• Although bystander cardiopulmonary resuscitation was statistically more likely in rural areas, 48-h survival improved over time in a few main cities of the canton of Vaud.

## Introduction

Out-of-hospital cardiac arrest (OHCA) is a significant cause of mortality in European countries.

A large multicentric European study conducted in 28 countries confirmed an mean incidence of 56 OHCAs per 100,000 inhabitants per year for patients considered for resuscitation, with 8% survival to hospital discharge in all cases with cardiopulmonary resuscitation (CPR) attempted.^1^ Considerable variations in OHCA incidence and outcome were observed across regions.^1^ In 2015, A Swiss study estimated an incidence of 73 OHCAs per 100,000 inhabitants per year treated by emergency medical services (EMS) in the Ticino canton.^2^

The incidence and outcome of OHCA depend on several elements not only related to patients and their cardiovascular risk factors,^3^ but also to contextual factors. The contributory role of the demographic and socioeconomic context on the incidence and outcome of OHCA has been highlighted^4^ and both may differ geographically within a country or region.^5^, ^6^ Geographic information systems (GIS) have been used to analyse these variations^7^, ^8^, ^9^ and explore the association between OHCA location and their regional incidence or outcome. Over the last decade, several Swiss studies have used similar spatial statistics to address specific public health issues, particularly in Geneva and Lausanne, and have demonstrated the significant spatial dependence of specific phenomena (e.g., high body mass index, mammography adherence or tobacco use).^10^, ^11^, ^12^

This study aimed to investigate the spatial dependence of incidence, bystander CPR (BCPR), and 48-h survival of OHCA in the canton of Vaud and if they were associated with specific demographic or socioeconomic characteristics. A comprehensive analysis of the effect of contextual factors on OHCA would allow for preventive interventions and adjustments of the current public health system.

## Methods

### Ethical approval

The study was approved by the local ethics committee (Commission cantonale d'éthique de la recherche sur l’être humain CER-VD, N° 20019-01286). Patient identity was coded and the research team did not have access to any identification data.

### Study design and population

We conducted a retrospective observational study based on the analysis of a clinical dataset including all consecutive, non-traumatic, EMS-attended OHCA patients in the Swiss canton of Vaud between 2007 and 2019. In 2019, the total population of Vaud was 806,088 (157,383 < 18 years [19.5%] and 126,217 > 65 years [15.7%]).^13^ The canton has a mixed geography of plains, rural and mountainous areas, with several densely populated urban regions located along Lake Geneva. Its health system comprises a university hospital, which serves as the regional hospital for the city of Lausanne (167,474 inhabitants in 2019) and adjacent boroughs, and six other regional, acute care hospitals.

A single emergency dispatch centre coordinates all the cantonal EMS. Trained paramedics constitute the initial response for out-of-hospital rescue missions. Prehospital emergency physicians may be dispatched by ambulance or helicopter for life-threatening emergencies or at the paramedics’ request. Depending on their protocols, paramedics may decide not to initiate cardiovascular resuscitation in the presence of obvious signs of death or “do-not-resuscitate” directives. Obvious signs of death include lividity, rigor mortis, decapitation, decomposition, crushing of the thorax, and significant loss of brain tissue. “Do-not-resuscitate” directives include written documents signed by the patient, directives reported in the medical record, or sufficient arguments in the heteroanamnesis. In case of uncertainty, paramedics act in the patient's best interest while waiting for medical reinforcement or a joint decision. Paramedics are allowed to consider resuscitation termination after 30 min of well-conducted resuscitation without return of spontaneous circulation (ROSC) or 20 min of persistent asystole only in the absence of the following circumstances: presence of life signs; any electrical tracing other than asystole; infants; pregnancy; hypothermia; drowning; or suspected intoxication. Additional resuscitative measures and transportation are indicated in these circumstances.

### Data definition and collection

Data were extracted from the cantonal health service database containing all types of out-of-hospital interventions in the canton. The latter constitutes mandatory medico-legal documentation for any out-of-hospital emergency intervention. The dataset comprised all rescue missions for non-traumatic OHCAs involving an EMS crew between 2007 and 2019, irrespective of whether the patient was resuscitated by EMS or if no action was taken. It included all-aged OHCAs of presumed cardiac or unknown aetiology. Each OHCA is coded in the database using a unique identifier. The exact geographical situation is recorded using the postal address of the intervention and the type of location (home, public place, retirement home). The dataset also contains information about the mission (date, hour, crew composition, duration, or delays) and the patient (identity, gender, age, presumed diagnosis). Additional data are collected for cardiac arrest patients according to Utstein recommendations.^14^ BCPR was defined as any resuscitation attempts by witnesses before the arrival of a professional rescue team. CPR by a professional rescuer was not considered as BCPR.

Local rates of cardiac arrest were calculated to identify regions at high risk of OHCA. All OHCA locations and attributes were aggregated within cantonal postcode areas. Population data compiled for these areas were based on the 2019 census (MicroGis Ltd, St Sulpice, Switzerland).^15^, ^16^ In the Lausanne urban area, a spatial analysis was conducted at the hectare level. This area comprises approximately 20% of the Vaud population, but with significant density variations. Appendix A contains geographical details of the Lausanne region and the definition used for cities vs. rural areas. Demographic and socioeconomic data from 2017 were used to characterise the hectare. Study demographic and socioeconomic variables included the proportion of the population aged 65 years or older, population density, proportion of males, ratio of Swiss citizens, and median salary income. Swiss citizens are defined as individuals who hold Swiss nationality, either by birth or through naturalisation.

### Statistical analysis

OHCA patients were characterised according to age, gender, intervention location, initial rhythm, witness status, ROSC and automated external defibrillator (AED) use, BCPR provision, and 48-h survival. The global incidence of cardiac arrest was calculated as the mean OHCA per 100,000 adjusted by age and sex during the study period. We evaluated OHCA incidence for each year from 2007 to 2019 using the corresponding population census, adjusted for age and sex, and calculated the corresponding 95% confidence interval. (Appendix B, Table B.1) For the spatial analysis of each OHCA, geocoding was performed using the mission destination address and postcode area with a Google Maps© API plugin (MMQGIS in QGIS software; Quantum GIS development team).^17^, ^18^ Addresses without street numbers were approximated to the centre of the street; if the address was missing, the OHCA location was approximated to the centroid of the postcode. Cases that could not be geocoded were excluded. After aggregation, three datasets were analysed: individual OHCA patients’ georeferenced points and attributes; OHCA aggregated at the postcode area level; and OHCA aggregated at the hectare level in the Lausanne urban area. OHCA incidence for postcode areas and hectares were calculated according to the 2019 population for the former and 2017 for the latter.

We used two spatial statistical approaches to explore OHCA possible spatial dependence, i.e., the Getis-Ord Gi statistic^19^ and the Local Moran’s I^20^ with the empirical Bayes (EB) standardised rate.^21^ With both methods, the null hypothesis asserts spatial randomness, and the significance test is determined by a conditional randomisation procedure of 999 permutations.^19^, ^20^ For map production, the level of statistical significance was set at p < 0.05. Individual patients, postcode areas, and hectares without neighbours (based on predetermined spatial lag [see Appendix A]) were excluded from the spatial analysis.

An individual canton-wide analysis of OHCA patients was performed by the Getis-Ord Gi method, which allowed a clear and robust overview to identify regions with statistically more BCPR provision and better 48-h survival.^19^ To exclude the main confounding factors and similar to other international publications^22^, the spatial analysis of BCPR only considered witnessed OHCAs. The spatial analysis of 48-h survival only considered patients resuscitated by bystanders. The Getis-Ord method^19^ is more straightforward than Local Moran's I. A value significantly higher than the mean indicates a hotspot (shown as a red dot), and a value significantly lower than the mean suggests a coldspot (shown as a blue dot). The terms “hotspots” and “coldspots” are used exclusively in analyses using the Getis-Ord method. The Getis-Ord approach does not consider spatial outliers.

Analysis of the incidence of OHCA aggregated by postcode area and by hectare requires a more detailed analysis to take outliers into account: even in a high-risk area, the presence of dissimilarity is likely. Using the Local Moran's I method is a more pragmatic approach to examining these phenomena. This robust method makes it possible in a single analysis to highlight risk areas (i.e., regions with spatial dependence) and reveal outliers (areas with dissimilar behaviour) within high- or low-risk clusters. As described by Anselin in 1995, the Local Moran's I method classifies observations into five well-defined classes based on Moran's scatterplot.^20^ In our study, the relationship between the incidence of OHCA and the mean incidence in a dedicated neighbourhood (spatial lag) was calculated for each postcode area or hectare. The Local Moran’s I method provides five different classes depending on the type of relationship between the local value and the mean value of the neighbourhood and a naming convention is clearly defined internationally: two classes with a spatial clustering of similar values (called “high-high” and “low-low” spatial clusters); two classes with spatial association between dissimilar values (“high-low” and “low–high” spatial outliers); and a neutral class without spatial dependence. Spatial outliers are values that demonstrate substantial dissimilarity in their spatial associations with neighbouring data points. Applied to our data, high-high clusters comprised high OHCA incidence postcode areas or hectares in a high OHCA incidence neighbourhood (and identically for low-low clusters). High-low outliers comprised high OHCA incidence postcode areas or hectares in a low OHCA incidence neighbourhood (and identically for low–high outliers).

Furthermore, we accounted for the high variability in population distribution between urban and rural areas. As the average number of cardiac arrests between each postcode area or hectare can be very volatile, the Local Moran’s I cluster map with empirical Bayes standardised rates was used instead of univariate Local Moran’s I cluster map to analyse the incidence of OHCA at these levels (see details in Appendix A).^21^ All spatial analyses were conducted using GeoDa software.^23^ Subsequent maps were created in QGIS.^24^ Background map layers were provided by MapTiler planet API^25^ using OpenStreetMap open-source data.^26^.

At the postcode area and hectare level, OHCA incidence was compared between high-high and low-low spatial clusters. Locations with spatial outliers (high-low and low–high outliers) appear on choropleth maps, but are excluded from the statistical analyses of demographic and socioeconomic characteristics. Discrete data were compared using a Chi2 test excluding missing values. Continuous data were compared using a Kruskal Wallis test excluding missing values. Statistical analyses were conducted with Stata 17.0 (StataCorp 2023 College Station, TX) and the level of statistical significance was set at p < 0.05.

## Results

Of 6,992 OHCAs listed in the cantonal database between 1 January 2007 and 31 December 2019, a total of 4,869 were included in our analysis (Appendix B, Fig. B.1). The mean incidence of EMS-attended OHCA with a presumed cardiac (or unknown) cause was 50.4 per 100,000 inhabitants per year, adjusted by age and sex according to the population census. EMS-attended OHCA incidence remained stable during the study period, with slightly lower rates in 2007 and 2008 (Appendix, Table B.1). OHCA patients had a median age of 71 years (interquartile range 59–81 years) and 67.9% were male (Table 1). Fifty-two patients of OHCA were < 18 years and 3,023 cases occurred in patients > 65 years. Three-quarters of OHCAs occurred in patients’ homes, followed by public places (19.8%). More than one-half of the initial rhythms were asystole, followed by shockable rhythms (21.3%), and pulseless electrical activity (12%). The overall BCPR rate was 41.8%, with lay witnesses present in 46% of cases; 13% of OHCAs occurred in the presence of healthcare or EMS providers.

**Table 1:** Characteristics of OHCA patients in the study population.

	All, n(%) n = 4869	With BCPR 2036 (41.8)	Without BCPR or unknown 2833 (58.2)	p-value	Alive at 48-hours	p-value
**Age (median, IQR)**	71 (59–81)	68 (56–78)	73 (61–83)		66 (54–78)	
**Sex**				p < 0.001*		p = 0.034*
Male	3308 (67.9)	1446 (43.7)	1862 (56.3)		563 (17.0)	
Female	1560 (32.1)	589 (37.8)	971 (62.2)		228 (14.6)	
Unknown	1 (0)	1 (100)	0		0 (0)	
**Location of intervention**				p < 0.001*		p < 0.001*
Home	3628 (74.5)	1331 (36.7)	2297 (63.3)		430 (11.9)	
Public place	838 (19.8)	467 (55.7)	371 (44.3)		262 (31.3)	
Retirement home	141 (2.9)	58 (41.1)	83 (58.9)		13 (9.2)	
Work and education place	118 (2.4)	71 (60.2)	47 (39.8)		36 (30.5)	
Sport and leisure facility	72 (1.5)	61 (84.7)	11 (15.3)		27 (37.5)	
Medical office	57 (1.2)	39 (68.4)	18 (31.6)		20 (35.1)	
Unknown	15 (0.3)	9 (60.0)	6 (40.0)		3 (20.0)	
**Initial rhythm**				p < 0.001*		p < 0.001*
Asystole	2716 (55.8)	1100 (40.5)	1616 (59.5)		189 (7.0)	
Ventricular fibrillation	963 (19.8)	624 (64.8)	339 (35.2)		414 (43.0)	
Pulseless electrical activity	586 (12.0)	204 (34.8)	382 (65.2)		104 (17.8)	
Ventricular tachycardia	74 (1.5)	27 (36.5)	47 (63.5)		36 (48.7)	
Unknown	530 (10.9)	81 (15.3)	449 (84.7)		48 (9.1)	
**Witness by**				p < 0.001**		p < 0.001*
Lay witnesses	2242 (46.0)	1225 (54.6)	1017 (45.4)		477 (21.3)	
No witness	1929 (39.6)	606 (31.4)	1323 (68.6)		90 (4.7)	
EMS crew	402 (8.3)	N/A	N/A		151 (37.6)	
Other healthcare provider	230 (4.7)	177 (77.0)	53 (23.0)		72 (31.3)	
Unknown	66 (1.4)	5 (7.6)	61 (92.4)		1 (1.52)	
**ROSC**				p < 0.001		p < 0.001
With ROSC	1179 (24.2)	630 (53.4)	549 (46.6)		761 (64.6)	
None or Unknown	3690 (75.8)	1406 (38.1)	2284 (61.9)		30 (0.8)	
**AED used**				p < 0.001		p < 0.001
AED used	204 (4.2)	201 (98.5)	3 (1.5)		61 (29.9)	
None or Unknown	4665 (95.8)	1835 (39.3)	2830 (60.7)		730 (15.7)	
**48-hours survival**				p < 0.001		
Alive	791 (16.3)	428 (54.1)	363 (45.9)		N/A	
Dead	4078 (83.7)	1608 (39.4)	2470(60.6)		N/A	

N/A (not applicable): analysis of the CPR bystander by the witness when the OHCA witness is the EMS crew is not relevant.

Significance tested by Chi2 test. *Excluding unknown data for analysis. ** Excluding unknown and irrelevant data (N/A).

IQR: interquartile range, BCPR: bystander cardiopulmonary resuscitation, ROSC: return of spontaneous circulation, AED: automated external defibrillator.

Without adjusting for underlying confounding factors, we found that shockable rhythms may be associated with better survival, the presence of witnesses (especially healthcare providers or paramedics) could improve survival, and AED use in 4.2% of cases appeared to have almost doubled 48-h survival. The ROSC rate was 24.2% and the overall 48-h survival rate was 16.3%. The 48-h survival rate could be increased for men (17.0% vs. 14.6%, respectively) and when OHCA occurred in public places, sports or work facilities, or the doctor’s office. Survival at 48-h seemed also better in the group that received BCPR (21.0% vs. 12.8%). However, additional studies are required to account for confounding factors and confirm all these results.

### Spatial dependence of BCPR and 48-h survival canton-wide

**Fig. 1:**
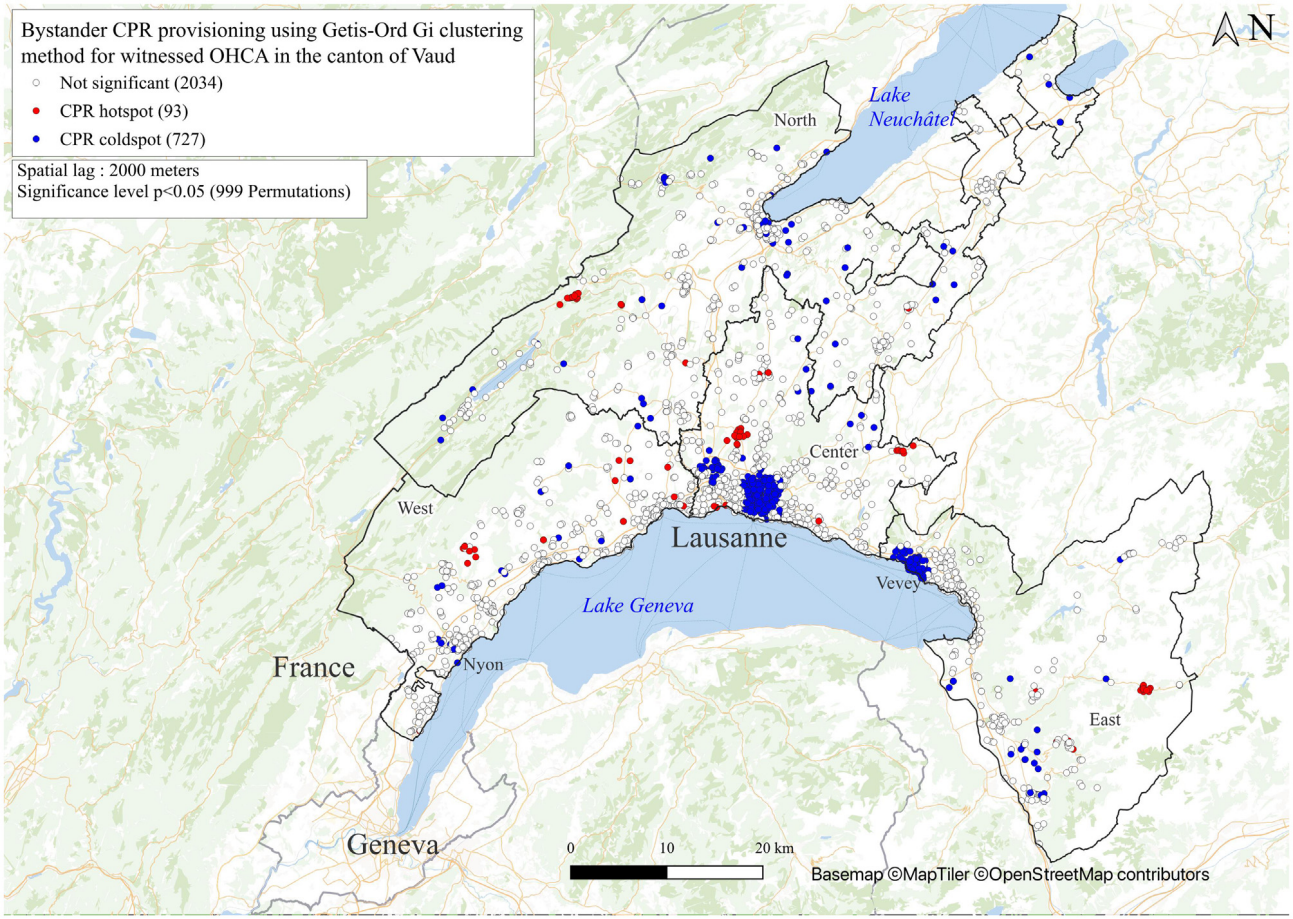
Bystander CPR (BPCR) provision using the Getis-Ord Gi clustering method for witnessed OHCA in the canton of Vaud. Red dots (hotspots) represent groups of patients with a BCPR provision mean significantly higher than the mean for the whole patient populations. Blue dots (coldspots) represent groups of patients with a BCPR provision mean significantly lower than the mean for the whole patient population. Neutral locations (without spatial dependence) are shown in white. Clustering takes into consideration the neighbours within a radius of 2000 m (spatial lag). Unwitnessed OHCA or without neighbours were excluded from the spatial analysis. (For interpretation of the references to colour in this figure legend, the reader is referred to the web version of this article.)

**Fig. 2:**
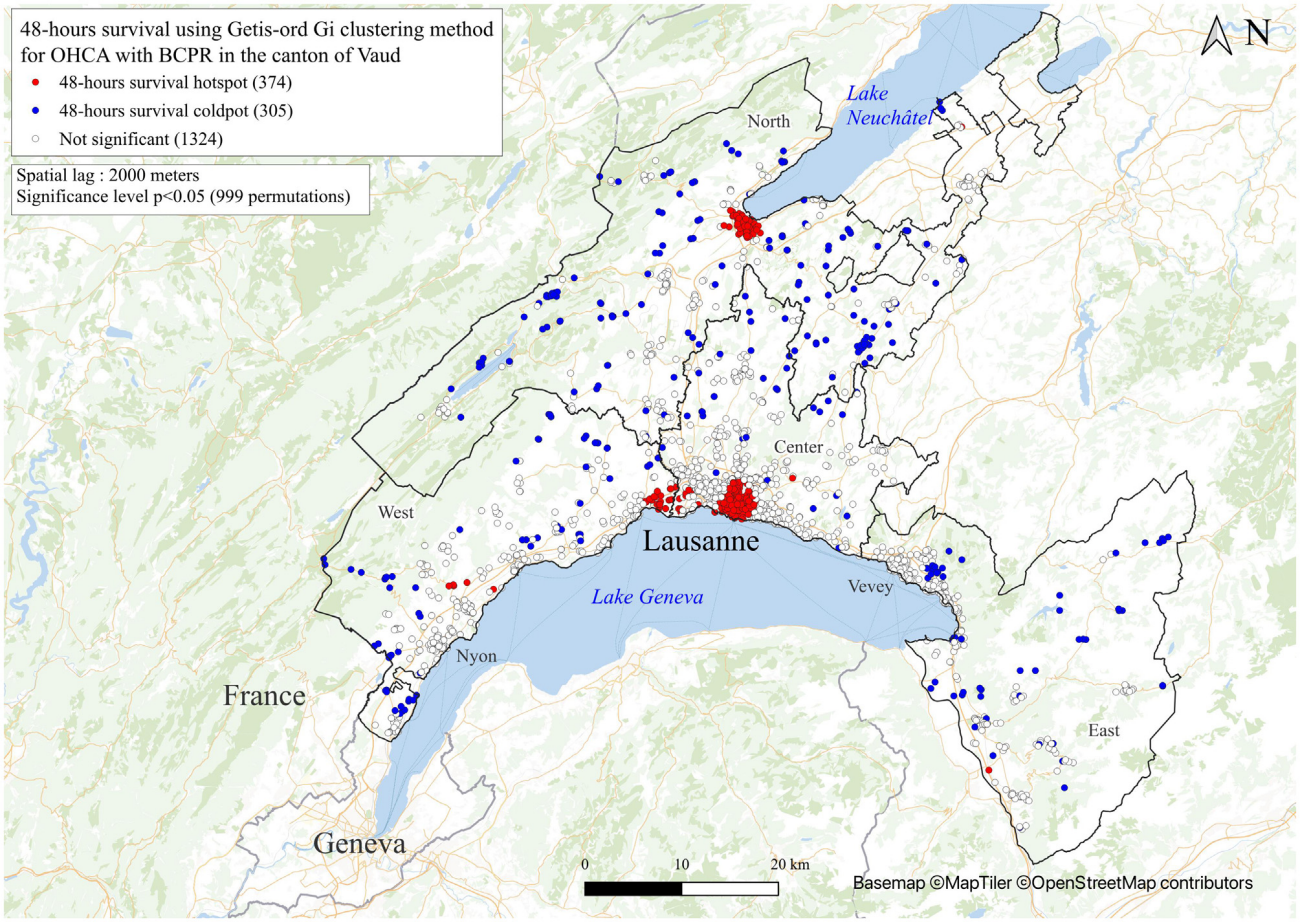
48-h survival using the Getis-Ord Gi clustering method for OHCA with BCPR in the canton of Vaud. Red dots (hotspots) represent groups of patients with a 48-h survival rate significantly higher than the mean for the whole patient population. Blue dots (coldspots) represent groups of patients with a 48-h survival rate significantly lower than the mean for the whole patient population. Neutral locations (without spatial dependence) are shown in white. The clustering considers the neighbours within a radius of 2000 m (spatial lag). OHCA without BCPR or without neighbours were excluded from the spatial analysis. (For interpretation of the references to colour in this figure legend, the reader is referred to the web version of this article.)

In the spatial analysis of BCPR provision for witnessed OHCA, coldspots were identified where little or no BCPR was performed (Fig. 1). Overall, BCPR was less frequently performed in urban areas along the coast of Lake Geneva and the southern shore of Lake Neuchâtel, but more often in rural areas (Fig. 1). Using the same approach when considering OHCA with BCPR, different survival hotspots were visualised canton-wide in the 48-h survival map (Fig. 2). Unlike BCPR, survival was higher in urban areas than in peripheral regions, with many rural villages displaying mortality hotspots. Analyses of BCPR and 48-h survival for all OHCAs at the cantonal level show similar results. (Appendix B, Fig. B.2-B.3). Small area variations in survival were also observed in 48-h survival maps for the Lausanne area (Appendix B, Fig. B.4).

### Spatial dependence of OHCA incidence according to cantonal postcode areas

**Fig. 3:**
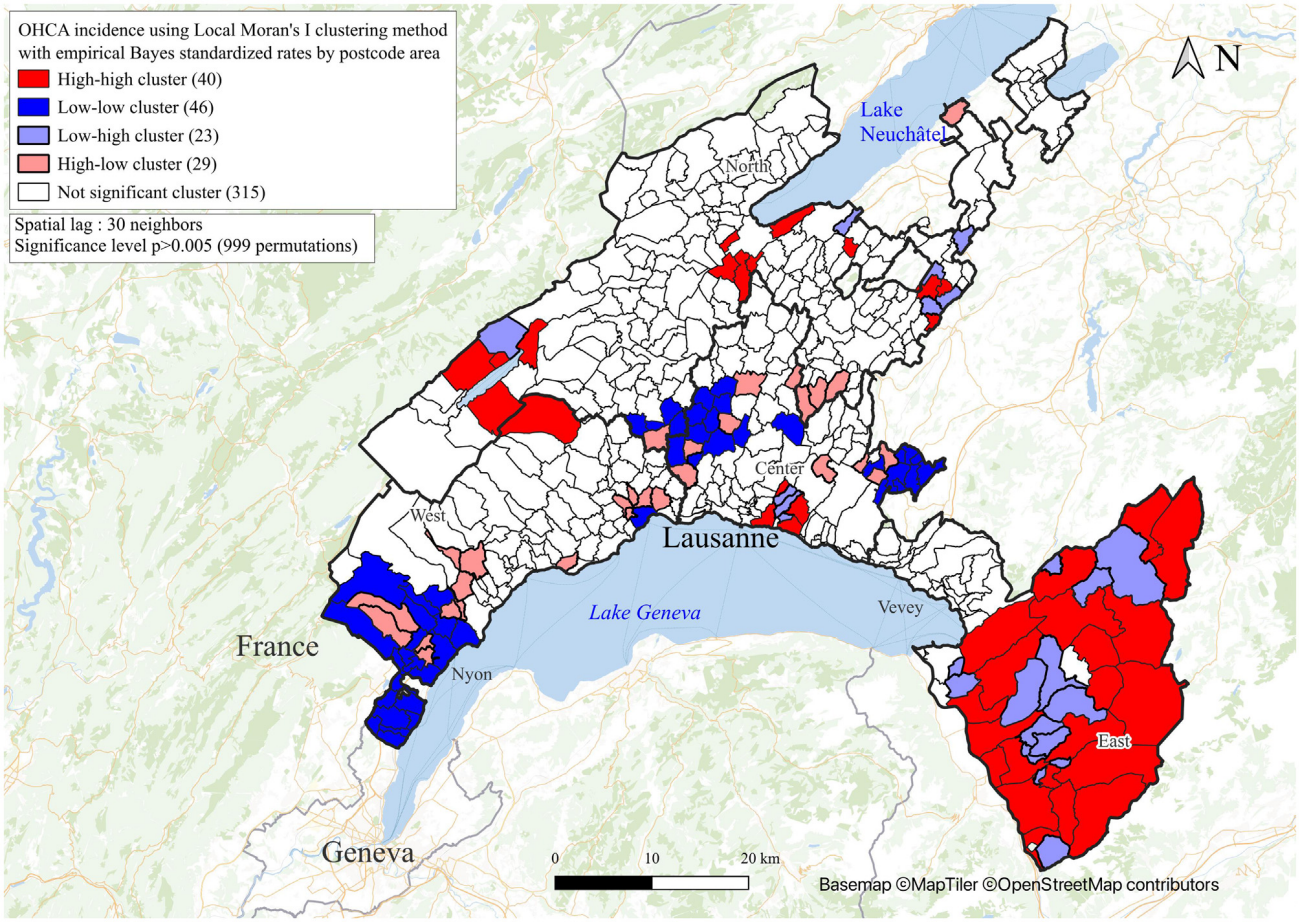
OHCA incidence by postcode area in the canton of Vaud. Dark red (high-high clusters) represent groups of postcode areas with OHCA incidence rates significantly higher in a high incidence neighbourhood. Dark blue (low-low clusters) represent groups of postcode areas with OHCA incidence rates significantly lower in a low incidence neighbourhood. Light blue (low–high outliers) represent groups of postcode areas with OHCA incidence rates significantly lower in a high incidence neighbourhood. Light red (high-low outliers) represent groups of postcode areas with OHCA incidence rates significantly higher in a low incidence neighbourhood. Neutral locations (without spatial dependence) are shown in white. The smoothing and clustering consider the 30 nearest neighbours (spatial lag). Postcode areas without neighbours were excluded from the spatial analysis. (For interpretation of the references to colour in this figure legend, the reader is referred to the web version of this article.)

OHCA incidence rates between postcode areas are presented in Fig. 3. Several high-high spatial clusters (i.e., groups of postcode areas with OHCA incidence rates significantly higher in a high incidence neighbourhood) were observed in the eastern area of the canton with many low–high spatial outliers (i.e., groups of postcode areas with OHCA incidence rates significantly lower in a high incidence neighbourhood), mainly located in tourist areas hosting mountain resorts. High-high clusters were found in the eastern boroughs of Lausanne and some areas in the east and north-west of the canton. The north-west Lausanne region had several low-low clusters (i.e., groups of postcode areas with OHCA incidence rates significantly lower in a low incidence neighbourhood) of OHCA.

A comparison of high-high and low-low clusters showed significant demographic and socioeconomic differences (Table 2). In postcode areas with high-high clusters, the population aged 65 years or older represented 20.5% vs. 15.1%, respectively. Median salary income was 24,200 Swiss francs lower in high-high clusters. Swiss citizens represented 83.4% in high-high clusters vs. 74.5% in low-low clusters. Finally, the population density was lower in high-high clusters compared to areas with a lower incidence: 0.6 vs. 2.9 inhabitants/hectare, respectively. There was no significant difference between genders.

**Table 2:** Comparison of demographic and socioeconomic factors between postcode areas in the canton of Vaud.

	High-high clusters n = 69	Low-low clusters n = 69	Not significant clusters n = 315	p-value
	
Age > 64 years ratio, median (IQR)	20.5% (±6.7%)	15.1% (±4.5%)	16.3% (±6.1%)	p < 0.001
Salary income in CHF, median (IQR)	56′800 (±16′100)	83′000 (±45′300)	66,200 (±16′800)	p < 0.001
Male ratio, median (IQR)	49.2% (±3.4%)	49.6% (±2.1%)	49.8% (±2.4%)	p = 0.42
Swiss nationality ratio, median (IQR)	83.4% (±12.0%)	74.5% (±16.6%)	83.3±% (±14.1%)	p = 0.026
Population density inhabitant/hectare, median (IQR)	0.63 (±1.2)	2.9 (±4.4)	1.1 (±2.3)	p < 0.001

Only high-high and low-low clusters determined by the local Moran’s I clustering method with empirical Bayes standardised rates were compared. High-high clusters represent groups of postcode areas with OHCA incidence rates significantly higher in a high incidence neighbourhood. Low-low clusters represent groups of postcode areas with OHCA incidence rates significantly lower in a low incidence neighbourhood.

The smoothing and clustering consider the 30 nearest neighbours (spatial lag). Postcode areas without neighbours are excluded from the spatial analysis.

Significance tested only between high-high and low-low clusters using the Kruskal Wallis test.

IQR, interquartile range, SD: standard deviation, CHF: Swiss franc.

### Spatial dependence of OHCA incidence in the Lausanne urban area

The same analysis was conducted in the Lausanne urban area and inhabited hectares were classified according to the OHCA incidence risk (Appendix B, Fig. B.5). An asymmetric distribution of OHCA rates was found as a peripheral belt around the Lausanne city centre where peripheral sectors adjacent to Lausanne were more affected than central hectares.

Socioeconomic variations between high-high and low-low clusters were observed (Table 3). The median monthly salary was higher in high-high clusters, i.e., 67,700 vs. 40,400 Swiss francs, respectively. Swiss citizens represented 83% in high-high vs. 60% in low-low clusters. High-high clusters also had a lower population density and included very slightly more males. There was no significant difference in the population aged 65 years or older between the two groups.

**Table 3:** Comparison of demographic and socioeconomic factors in the Lausanne region.

	High-high clusters n = 117	Low-low clusters n = 1608	Not significant clusters n = 3571	p-value
	
Age > 64 years ratio, median (IQR)	18.0% (±29.0%)	19.0% (±12.0%)	19.0% (**±**21.0%)	p = 0.149
Salary income in CHF, median (IQR)	67′700 (±21′900)	40′400 (±18′900)	58,000 (±21′800)	p < 0.001
Male ratio, median (IQR)	50.0% (±8.0%)	49.0% (±7.0%)	50.0% (±7.0%)	p = 0.024
Swiss nationality ratio, median (IQR)	83.0% (±32.0%)	60.0% (**±**25.0%)	76.0% (±35.0%)	p < 0.001
Population density inhabitant/hectare, median (IQR)	14.0 (±18.0)	88.0 (±122.5)	19.0 (±33.0)	p < 0.001

Only high-high clusters versus low-low clusters determined by the local Moran’s I clustering method with empirical Bayes standardised rates were compared.

High-high clusters represent groups of hectares with OHCA incidence rates significantly higher in a high incidence neighbourhood. Low-low clusters represent groups of hectares with OHCA incidence rates significantly lower in a low incidence neighbourhood.

The smoothing and clustering consider the neighbours within a radius of 800 m (spatial lag). Hectares without neighbours are excluded from the spatial analysis.

Significance tested only between high-high and low-low clusters using the Kruskal Wallis test.

IQR, interquartile range, SD: standard deviation, CHF: Swiss franc.

## Discussion

Our study confirms spatial variations in OHCA incidence and survival related to demographic and socioeconomic factors in the canton of Vaud. To our knowledge, no study of OHCA has analysed the spatial distribution of cardiac arrests in Switzerland, their short-term prognosis, and geographical locations. Epidemiological characteristics of OHCA in the canton were similar to international publications and corroborated a recent Swiss study analysing OHCA during the COVID-19 pandemic.^27^ Typically, OHCAs mainly affect men; they mostly take place at home, without a lay witness. The initial rhythm is often non-shockable.

BCPR was more likely in rural areas, but survival was better in a few main cities. Clusters of high OHCA incidence were observed at the cantonal level and in the Lausanne area, but the demographic and socioeconomic analyses revealed very different results.

We determined an annual incidence of 50.4 EMS-attended OHCA with a presumed cardiac cause per 100,000 inhabitants after adjusting for age and sex. However, these findings cannot be compared with incidences reported in previously mentioned European studies^1^, ^2^ as they considered EMS-treated (resuscitated) OHCA of all aetiologies. A 2008 study^28^ based on the Resuscitation Outcomes Consortium in 10 North American sites found that the incidence of EMS-attended OHCA of cardiac origin was 95.7 per 100,000. Further studies are needed to investigate the important gap reported between Switzerland and North America. In the future, the use of similar denominators for European cohorts and Swiss EMS-treated OHCAs of all aetiologies will allow for reliable comparisons.

Several studies have confirmed geographical variations in the incidence of OHCA.^7^, ^29^ The population age is probably an essential determinant of these variations,^30^ which was statistically older in high-high postcode clusters. Age as a determinant of OHCA incidence could also explain the spatial outliers found in the eastern region of the canton (low–high outliers) as some of these are major tourist areas, attracting a younger and healthier population. Geographic variations were also associated with the socioeconomic level.^5^, ^6^, ^31^, ^22^ High-high postcode clusters had a lower income and a higher proportion of non-Swiss citizens, thus confirming a 2019 *meta*-analysis showing an increased risk of OHCA, a decreased chance of receiving BCPR, and a decreased chance of survival in low socioeconomic areas.^32^

In the per hectare analysis of the Lausanne region, almost all high-high clusters were located outside the city centre in mainly residential neighbourhoods. By contrast to the cantonal-level analysis, these clusters had a higher salary income and a higher proportion of Swiss citizens, but no statistically different population age. To the best of our knowledge, no research has examined the spatial variations in OHCA at the hectare level in a city. The origin of these differences in the distribution of socioeconomic characteristics remains unclear. Factors such as the heterogeneity of population distribution, lifestyle, pollution,^33^ and socioeconomic levels between city centres and suburbs, as well as the influence of commuters, could play a role. Additional research into small-area variations in the city are therefore needed.

An association between population density and OHCA incidence was found at both cantonal and Lausanne levels: the lower the population density, the higher the OHCA incidence. Confounding factors such as socioeconomic conditions, demographics and cardiovascular risk factors were probably not unevenly distributed between high vs. low population density areas. However, socio-demographic studies analysing the spatial distribution of these factors are lacking in Switzerland. The BCPR rate tends to be inversely associated with population density.^22^, ^34^ Our geographical analyses also confirmed this pattern, with a predominance of coldspots in towns and cities. The greater distance to the hospital may encourage BCPR given the longer response times by paramedics, and a greater social connection may encourage bystanders to perform BCPR.^35^ Alternatively, the lower BCPR rate in more densely populated areas may reflect the “bystander effect”, referring to the inaction of bystanders in an emergency, expecting someone else to take the necessary action.^36^ Our BCPR rate was only 41.8%, while it was 58% in Europe between 2017 and 2019.^1^ However, it has been steadily increasing in Switzerland,^2^ partly due to the implementation of telephone-guided CPR and the involvement of first responders by the dispatch centre.^37^

The survival rate at hospital discharge was 8% in the EuReCaTwo study, while our 48-h survival rate was moderately higher. However, the rate comparison is limited as the time points were different as survival only considered EMS-treated cases in EuReCaTwo. Similar to reports in the literature,^38^ we observed a higher incidence of OHCA in men, but higher mortality in women. We also observed a potentially better prognosis in cities than in rural areas.^39^, ^40^ Moreover, when looking at the distribution of high survival hotspots in Lausanne Appendix B, Fig. B.4, there was an inverse relationship with the distance to Lausanne University Hospital. Better access to hospitals and emergency departments, proximity to EMS systems, and shorter intervention times should play a major role in OHCA survival.

Our findings point to potential targeted interventions in high-risk areas in the canton of Vaud. Education in the early detection of cardiac arrest, basic life support training, prevention of cardiovascular risk factors, provision of additional AEDs in specific locations, and further recruitment of first responders could improve patient prognosis. The BCPR rate must be improved, particularly in the main cities of the canton. Finally, populations with a lower socioeconomic level should also be the target of these specific interventions to prevent and mitigate the consequences of cardiovascular diseases.

### Strengths and limitations

The main strength of the study is the use of powerful and robust methods to analyse the spatial dependence of OHCA in the canton of Vaud with a fine granularity, which allowed to identify variations associated with demographic and socioeconomic characteristics. A second strength is the use of the cantonal database, which documents all OHCAs for which an EMS was dispatched. However, our study has some limitations. First, our retrospective analysis relied on routinely-collected data whose exhaustivity and quality was variable. Notably, intervention form completion was not standardised between paramedics, leading to missing values and approximations, similar to OHCA variations reported internationally.^41^ We included all OHCAs involving a prehospital rescue team, regardless of whether the patient was resuscitated or not, due to the lack of EMS-treated OHCA data reported between 2007 and 2010. Thus, we could not compare the incidence of OHCA in our sample with European data. Data for spatial analyses were aggregated over 13 years, which may have resulted in a temporal effect. However, this effect is presumably negligible as the incidence of OHCA remained relatively stable over the study period (apart from slightly lower rates in 2007 and 2008). Furthermore, the availability of detailed socioeconomic and sociodemographic data related to the postcode area, especially at the hectare level, was not annual and restricted to specific years according to the federal census. Therefore, we used population count, socioeconomic and sociodemographic characteristics based on the 2017 and 2019 census data for hectares and postcode areas, respectively. These characteristics may have possibly varied over time. Approximately 25% of OHCAs occurred outside the patient’s home. Therefore, associations between high-risk areas or neighbourhood-level factors and OHCA were prone to approximations. This issue may affect preferentially cities where a substantial proportion of the population works or commutes. Finally, we did not consider post-OHCA medical management, a critical determinant of 48-h survival.

## Conclusions

This retrospective study shows that a better understanding of factors influencing OHCA is essential to reduce its incidence. Improvement of the quality of data collection during rescue missions is a first step to improve the accuracy of analyses performed on OHCAs. Further studies are needed to confirm whether these urban vs. rural and city centre vs. periphery differences exist elsewhere in Europe and their underlying mechanisms. Our findings are valuable to support evidence-informed policymaking by public health authorities or politicians for the introduction of targeted interventions in areas at higher risk of OHCAs and to reduce the burden in the canton of Vaud.

## CRediT authorship contribution statement

**Guillaume Lengen:** Conceptualization, Investigation, Methodology, Writing – original draft, Writing – review & editing. **Olivier Hugli:** Conceptualization, Methodology, Supervision, Writing – review & editing. **David De Ridder:** Conceptualization, Investigation, Methodology, Writing – review & editing. **Idris Guessous:** Conceptualization, Investigation, Methodology, Validation, Writing – review & editing. **Anaïs Ladoy:** Investigation, Methodology, Validation, Writing – review & editing. **Stéphane Joost:** Conceptualization, Methodology, Supervision, Validation, Writing – review & editing. **Pierre-Nicolas Carron:** Conceptualization, Supervision, Writing – original draft, Writing – review & editing.

## Declaration of competing interest

The authors declare that they have no known competing financial interests or personal relationships that could have appeared to influence the work reported in this paper.
