# Supplementary material for: Spatial dependence of non-traumatic out-of-hospital cardiac arrest in a Swiss region: A retrospective analysis
Source: Resusc Plus. 2024 Jul 13;19:100713. doi: 10.1016/j.resplu.2024.100713 (PMC11299561; doi:10.1016/j.resplu.2024.100713)
Supplement: Supplementary Data 1 [file mmc1.docx]

**Appendices**

# Appendix A. Geographical and methodological precisions

# City and rural area definition

The definition of cities on which this work is structured is detailed by the Swiss Federal Statistical Office.^*^ A "statistical city" must satisfy defined criteria (population density, presence of workplaces and overnight hotel stays). The relevant Vaud cities for our study are Lausanne, Montreux, Nyon and Yverdon-les-Bains. Any out-of-hospital cardiac arrest (OHCA) outside these cities is considered to have occurred in a rural area.^[[1]](#footnote-1)^*^*^

Lausanne region definition

In our analysis, the Lausanne region is mainly determined using the boundaries of the "Lausanne-Morges agglomeration project,"^**^ defined by the canton of Vaud. This area includes Lausanne at its core and extends from Morges to Lutry (West-East) and Cheseaux-Sur-Lausanne to Lake Geneva (North-South). The "Lausanne-Morges agglomeration project" has divided the area into five sectors, with a central sector known as the Lausanne centre (a highly urbanised area) and four more peripheral sectors, namely the North, East and West of Lausanne, and the Morges region. Our manuscript uses the same terminology and compares the Lausanne city centre (the Lausanne centre region that includes the municipalities of Lausanne and Epalinges) with the more peripheral sectors.

Local spatial autocorrelation methods: technical precisions

We used two spatial statistical approaches to explore the possible spatial dependence of OHCA: the Getis-Ord Gi statistic^19^ and the Local Moran’s I^20^ with the empirical Bayes (EB) standardised rate to generate a cluster map.^21^ These tools highlight geospatial clusters expressing the phenomenon’s spatial arrangement by supposing that neighbouring objects often display similar behaviours. The Local Moran’s I cluster map is based on the global Moran’s I statistic^***^; the Getis-Ord Gi statistic is a Z-score.^19^ They compare an attribute at a given location to the same attribute values in its neighbouring location (spatial lag). We used a spatial lag of 2000 m at the cantonal scale and 800 m at the hectare level after testing several different spatial lags (200, 400, 600, 800, 1000 and 2000 m). For the Lausanne region, we used the same spatial lag as some previous geospatial studies in the canton.^8^ At the cantonal level, we considered the impact of greater distances due to a lower population density and therefore used a greater spatial lag. Finally, at the postcode level, it was more relevant to take into consideration the 30 nearest communes rather than a distance, due to the heterogeneity of commune sizes.

Using a 999 permutation, Gi significance maps (Appendix B, Figs. B.6 and B.7) illustrate local Gi statistics for every OHCA with its degree of significance, considering BCPR provisioning or 48-h survival spatial autocorrelation.

**Use of standardised EB rates**

The high variability in population distribution between urban and rural areas was accounted for. As the average number of cardiac arrests between each postcode area or hectare can be very volatile, the Local Moran’s I cluster map with EB standardised rates was used instead to analyse the incidence of OHCA at these levels. In general, the conventional EB smoothed rate method allows to calculate a rate or incidence smoothed by population size variability (also called ‘shrinkage estimators’) to take into consideration volatile rates due to this variability. The Local Moran’s I with standardised EB rates is comparable, but does not use smoothed rates. Instead, it generates a transformed (so-called “standardised”) randomised variable based on its mean and variance for each observation and then compares it with adjacent neighbours. This technique also overcomes the problems of variance instability.

# Classical EB-smoothed cardiac arrest incidence rates for postcode areas and the Lausanne hectare level are described in Appendix B, Figs B.8 and B.9.

# Appendix B

# Supplementary data

#

**Table B.1. Evolution of EMS-attended OHCA adjusted by age and sex over the study period.**

| Year | Residential population  in the canton of Vaud | Crude EMS-attended OHCA incidence  per 100,000 inhabitants | Adjusted EMS-attended OHCA incidence per 100,000 inhabitants | 95%  confidence interval |
| --- | --- | --- | --- | --- |
| 2007 | 668’581 | 40.7 | 41.5 | (36.7-46.8) |
| 2008 | 684’922 | 41.3 | 43.4 | (38.5-48.8) |
| 2009 | 697’802 | 46.1 | 46.9 | (41.9-52.3) |
| 2010 | 708’177 | 53.2 | 55.0 | (49.6-60.9) |
| 2011 | 721’561 | 53.2 | 53.6 | (48.4-59.3) |
| 2012 | 729’971 | 51.5 | 53.4 | (48.1-59.1) |
| 2013 | 743’317 | 51.5 | 51.3 | (46.3-56.8) |
| 2014 | 755’369 | 50.7 | 48.9 | (44.1-54.0) |
| 2015 | 767’497 | 55.5 | 54.1 | (49.1-59.6) |
| 2016 | 778’251 | 50.8 | 50.2 | (45.3-55.4) |
| 2017 | 794’384 | 54.4 | 54.1 | (49.1-59.4) |
| 2018 | 800’162 | 53.4 | 51.6 | (46.8-56.8) |
| 2019 | 806’088 | 50.6 | 49.8 | (45.0-54.8) |

OHCA: out-of-hospital cardiac arrest; EMS: emergency medical service.

**Fig. B.1.** Consort flow diagram

| OHCA in the Vaud cantonal dataset (2007-2019) n=6,992 OHCA | |  |
| --- | --- | --- |
|  |  |  |
|  |  | Excluded: 239 OHCA with a postcode in another canton or without EMS intervention |
|  |  |  |
|  |  |  |
| EMS-attended OHCA with a cantonal postcode n=4,944 OHCA | |  |
|  |  |  |
|  |  | Excluded :  1,670 traumatic or hypoxaemic OHCA |
|  |  |  |
|  |  |  |
| EMS-attended OHCA of presumed cardiac aetiology n=5,083 OHCA | |  |
|  |  |  |
|  |  | Excluded:  139 cases with missing or erroneous postal addresses |
|  |  |  |
|  |  |  |
| Geolocated EMS-attended OHCA of presumed cardiac aetiology n=4944 OHCA | |  |
|  |  |  |
|  |  | Excluded: 75 addresses geolocated outside the boundaries of the canton |
|  |  |  |
|  |  |  |
| Study population n=4869 OHCA | |  |

OHCA: out-of-hospital cardiac arrest; EMS: emergency medical service.

**Fig. B.2.** Bystander cardiopulmonary resuscitation (BPCR) provision using the Getis-Ord Gi clustering method for all out-of-hospital cardiac arrest (OHCA) in the canton of Vaud (witnessed and unwitnessed).


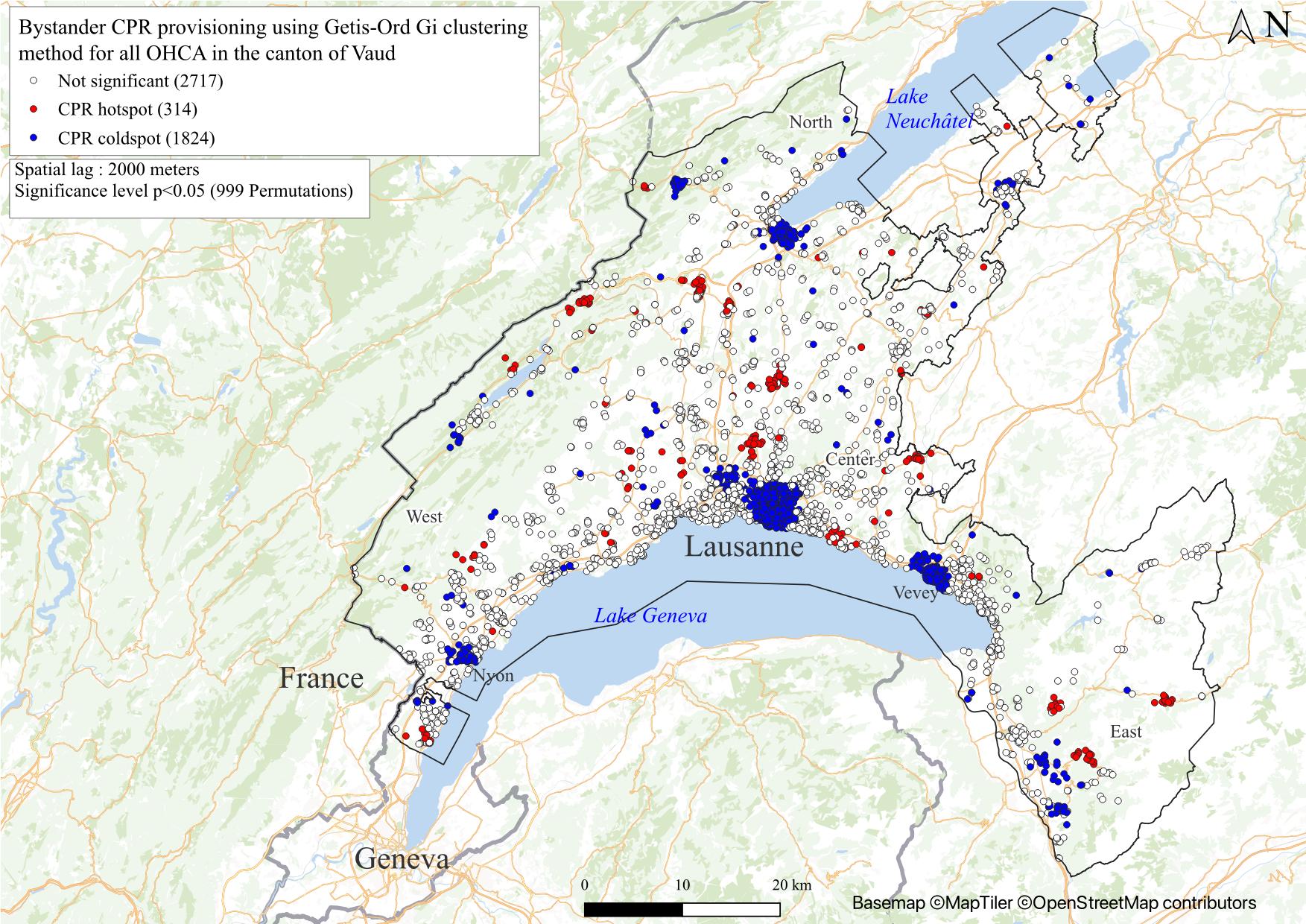


Red dots (hotspots) represent groups of patients with a BCPR provision mean significantly higher than the mean for the whole patient population. Blue dots (coldspots) represent groups of patients with a BCPR provision mean significantly lower than the mean for the whole patient population. Neutral locations (without spatial dependence) are shown in white. Clustering takes into consideration the neighbours within a radius of 2000 m (spatial lag). Patients without neighbours are excluded from the spatial analysis.

**Fig. B.3.** 48-h survival using the Getis-Ord Gi clustering method for all out-of-hospital cardiac arrest (OHCA) in the canton of Vaud (with and without bystander cardiopulmonary resuscitation [BCPR]).


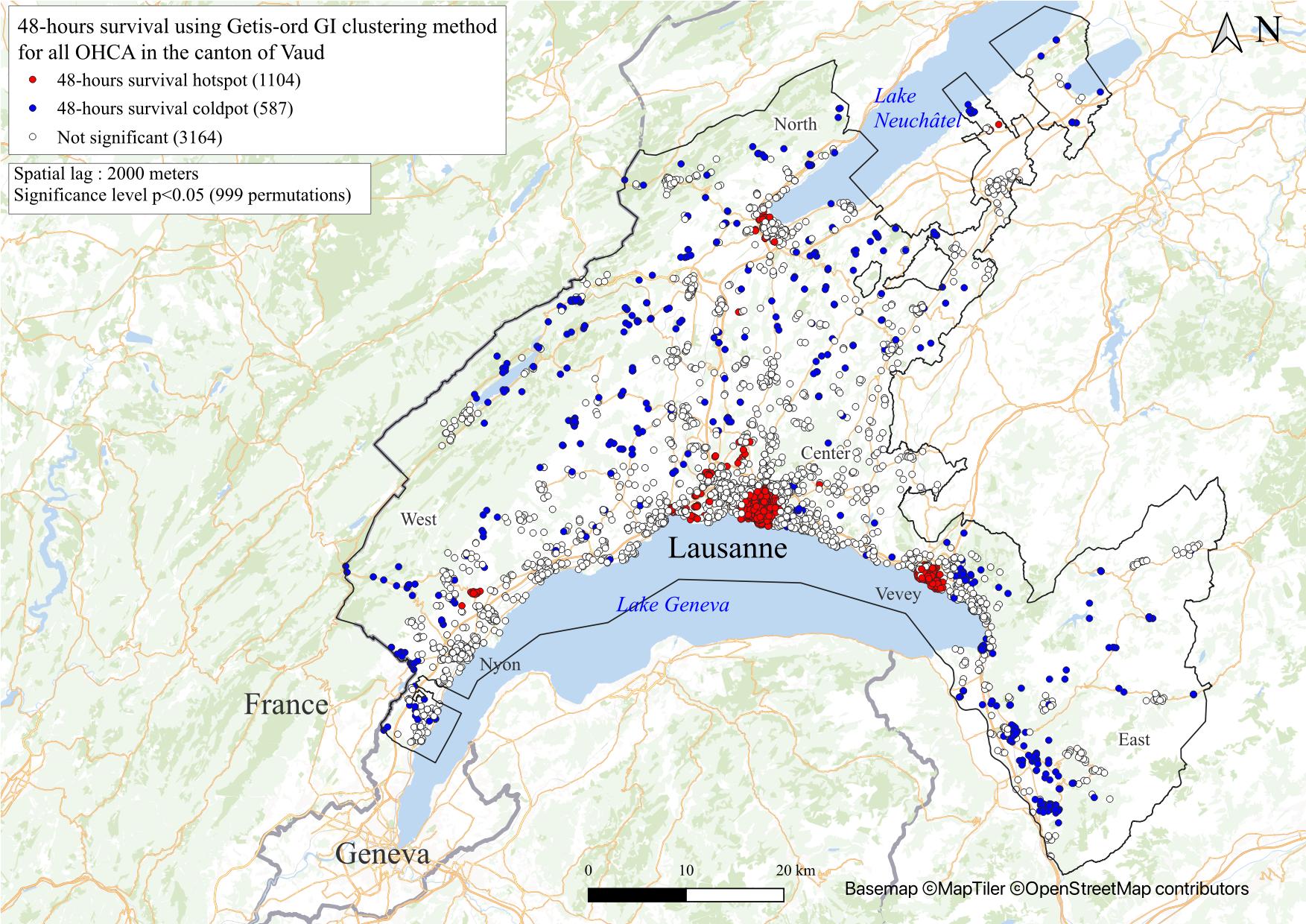


Red dots (hotspots) represent groups of patients with a 48-h survival rate significantly higher than the mean for the whole patient population. Blue dots (coldspots) represent groups of patients with a 48-h survival rate significantly lower than the mean for the whole patient population. Neutral locations (without spatial dependence) are shown in white. The clustering considers the neighbours within a radius of 2000 m (spatial lag). Patients without neighbours are excluded from the spatial analysis.

**Fig. B.4.** 48-h survival using the Getis-Ord Gi clustering method for all out-of-hospital cardiac arrests (OHCA) in the Lausanne area (with and without bystander cardiopulmonary resuscitation [BCPR]).


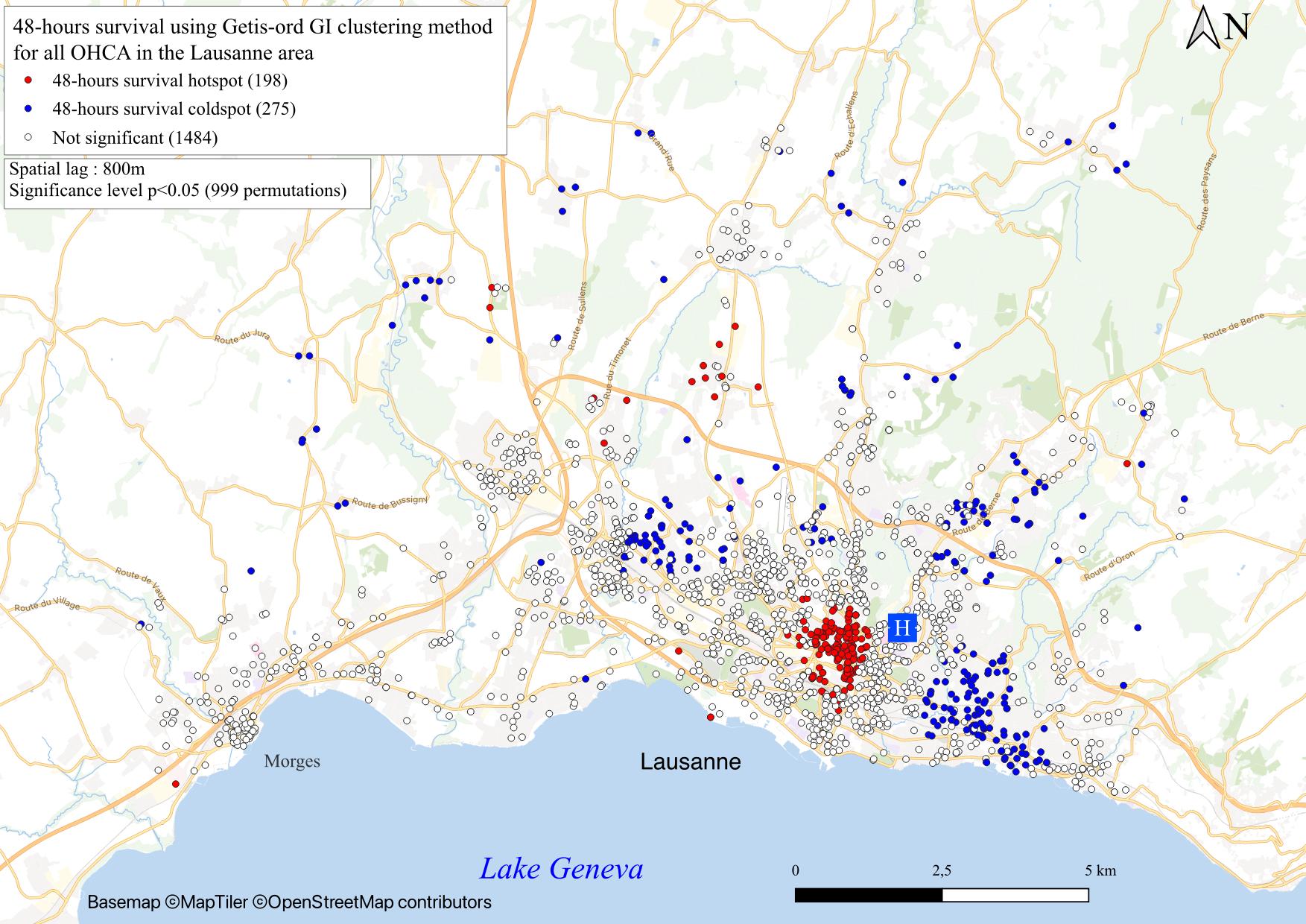


Red dots (hotspots) represent patients forming groups with a 48-h survival rate significantly higher than the mean of the whole population. Blue dots (coldspots) represent patients forming groups with a 48-h survival rate significantly lower than the mean of the whole population. Neutral locations (without spatial dependence) are shown in white. Clustering considers the neighbours within a radius of 750 m (spatial lag). The H symbol indicates the Lausanne University Hospital. Patients without neighbours are excluded from the spatial analysis.

**Fig B.5. Out-of-hospital cardiac arrest (OHCA) incidence in the Lausanne urban area.**


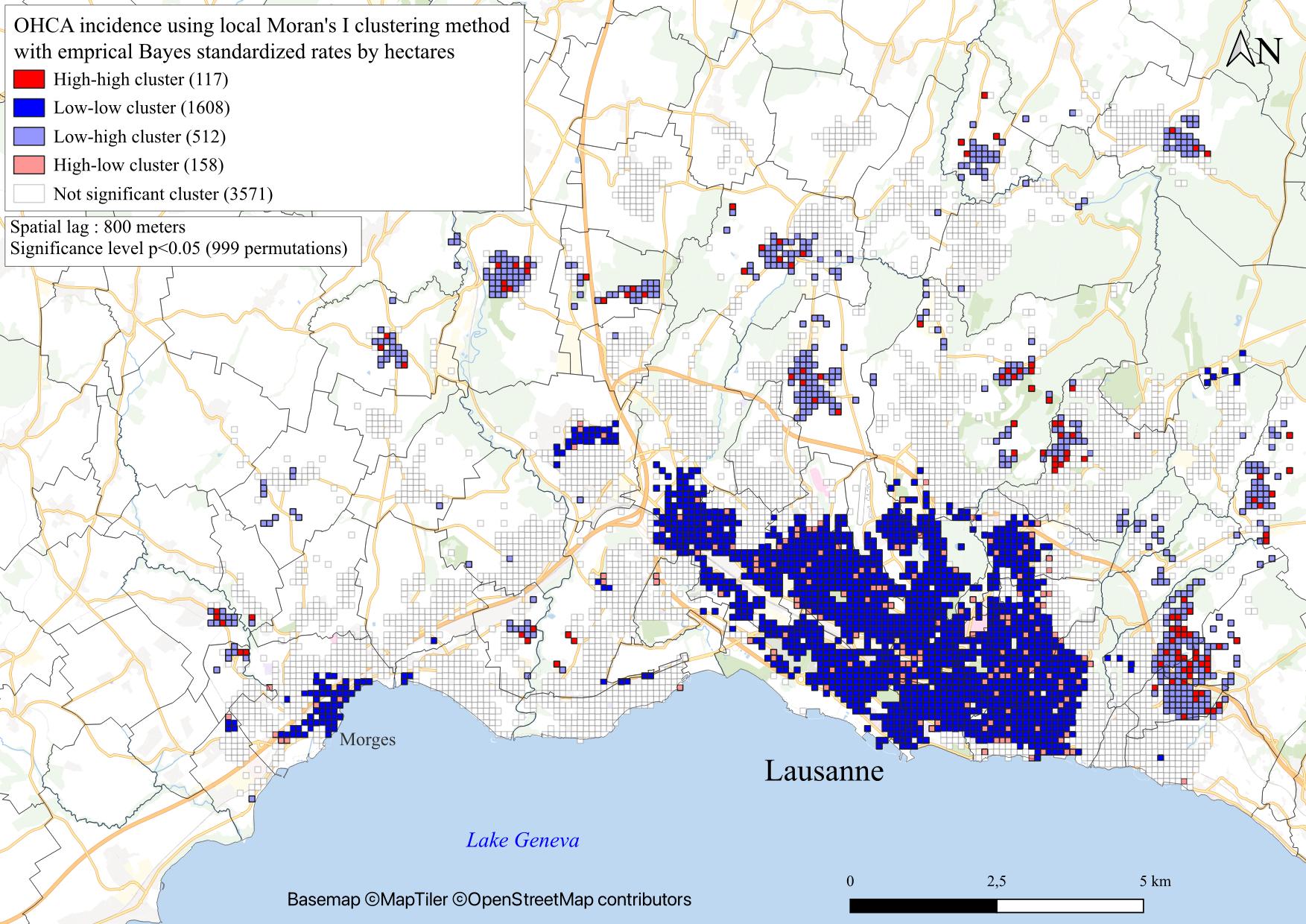


Dark red (high-high clusters) represents groups of hectares with OHCA incidence rates significantly higher in a high incidence neighbourhood. Dark blue (low-low clusters) represents groups of hectares with OHCA incidence rates significantly lower in a low incidence neighbourhood. Light blue (low-high outliers) represents groups of hectares with OHCA incidence rates significantly lower in a high incidence neighbourhood. Light red (high-low outliers) represents groups of hectares with OHCA incidence rates significantly higher in a low incidence neighbourhood. Neutral locations (without spatial dependence) are shown in white. The smoothing and clustering consider the neighbours within a radius of 800 m (spatial lag). Hectares without neighbours are excluded from the spatial analysis.

**Fig. B.6.** Gi significance map for bystander cardiopulmonary resuscitation (BCPR) provisioning for witnessed-out-of-hospital cardiac arrest (OHCA) in the canton of Vaud.


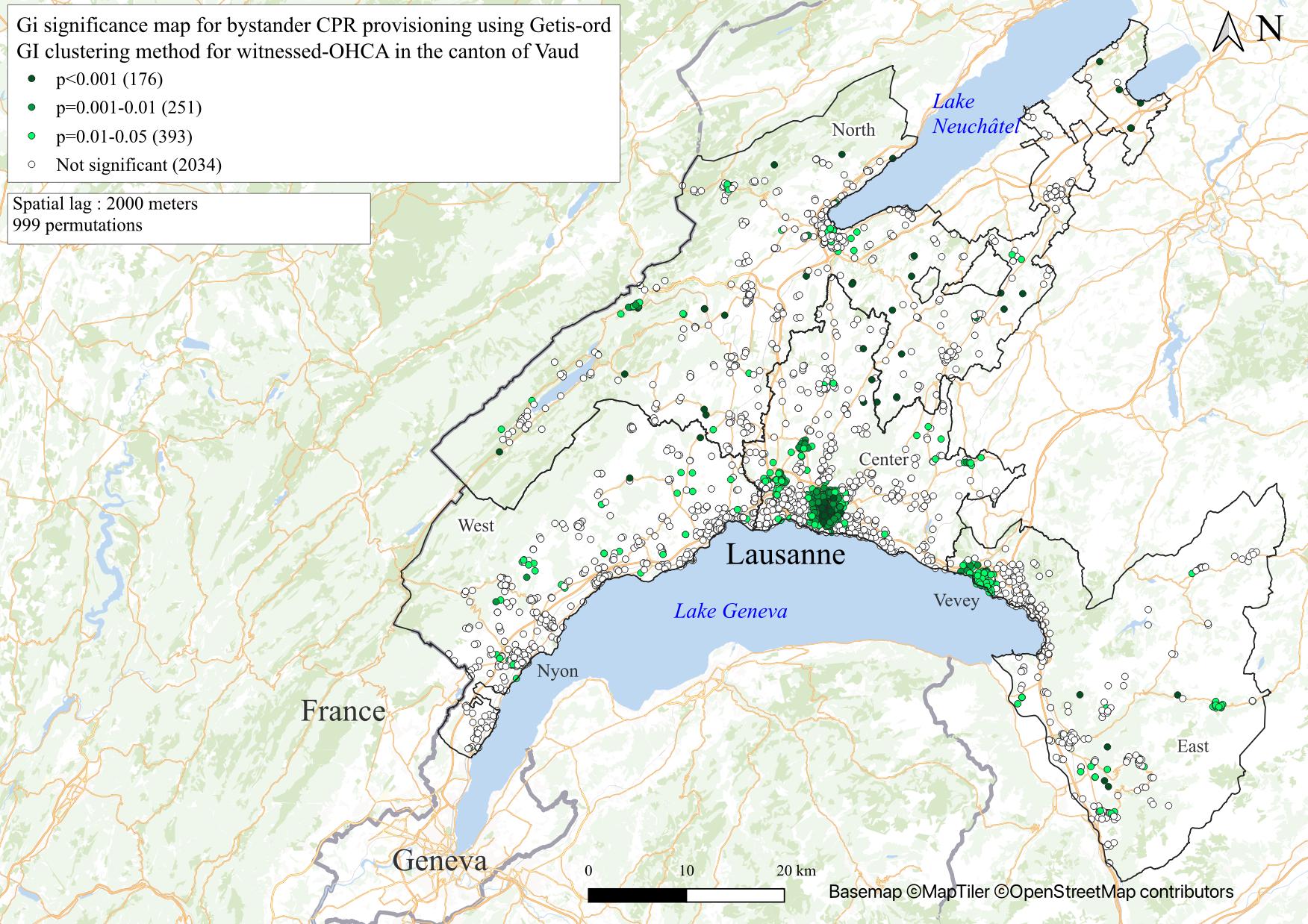


Using a 999 permutation, the Gi significance map illustrates local Gi statistics for every OHCA with its degree of significance, considering BCPR provisioning spatial autocorrelation. Light green shows a significance level between 0.01 and 0.05, medium green between 0.01 and 0.001, and dark green inferior or equal to 0.001. Neutral locations (without spatial dependence) are shown in white. Clustering considers the neighbours within a radius of 2000 m (spatial lag). Unwitnessed OHCA or without neighbours are excluded from the spatial analysis.

**Fig. B.7.** Gi significance map for 48-hours survival for out-of-hospital (OHCA) with bystander cardiopulmonary resuscitation (BCPR) in the canton of Vaud.


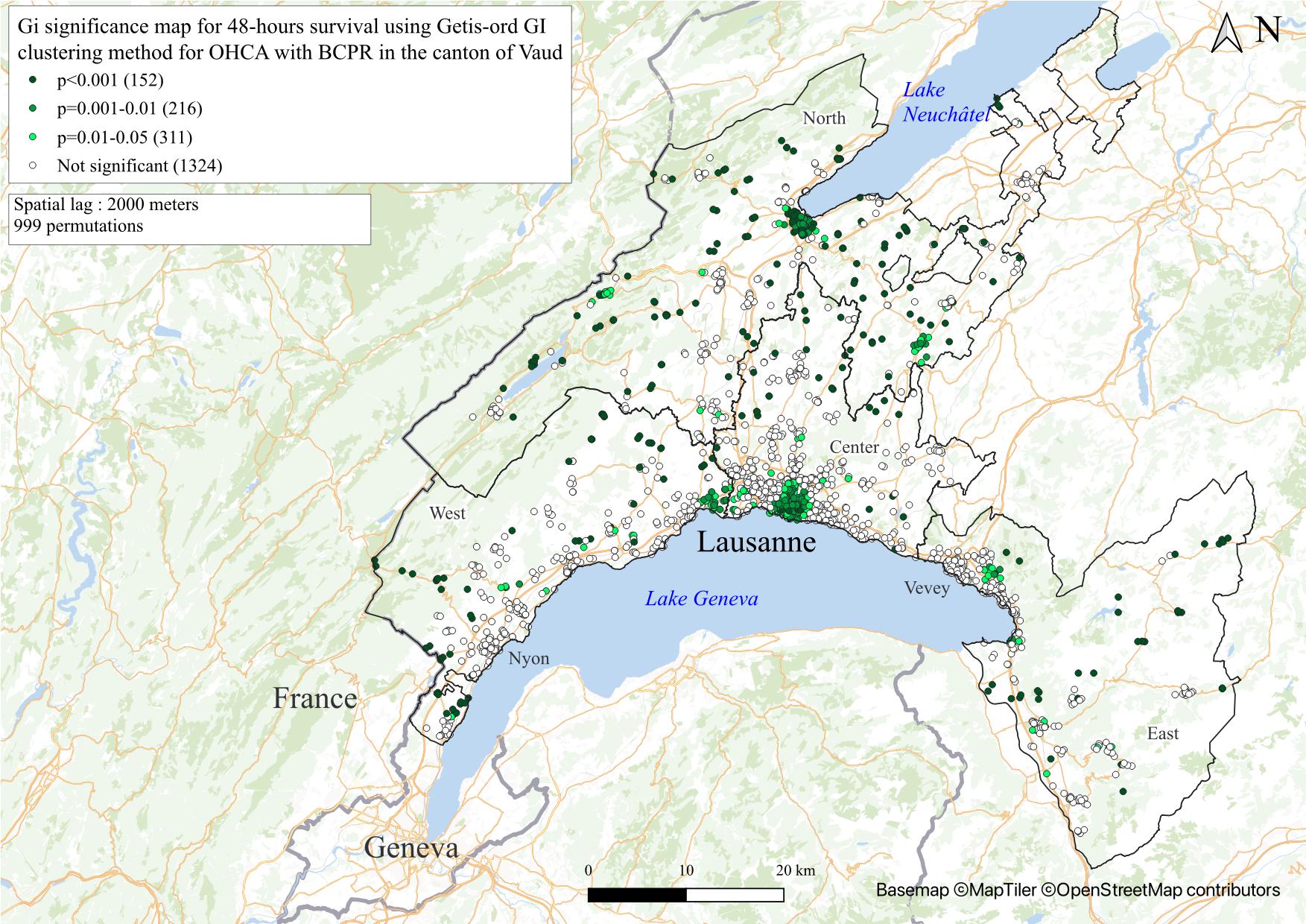


Using a 999 permutation, the Gi significance map illustrates local Gi statistics for every OHCA with its degree of significance, considering 48-h survival spatial autocorrelation. Light green shows a significance level between 0.01 and 0.05, medium green between 0.01 and 0.001m and dark green inferior or equal to 0.001. Neutral locations (without spatial dependence) are shown in white. Clustering considers the neighbours within a radius of 2000 m (spatial lag). OHCA without BCPR or without neighbours are excluded from the spatial analysis.

**Fig. B.8.** Empirical Bayes-smoothed out-of-hospital (OHCA) incidence rates in the Lausanne area.


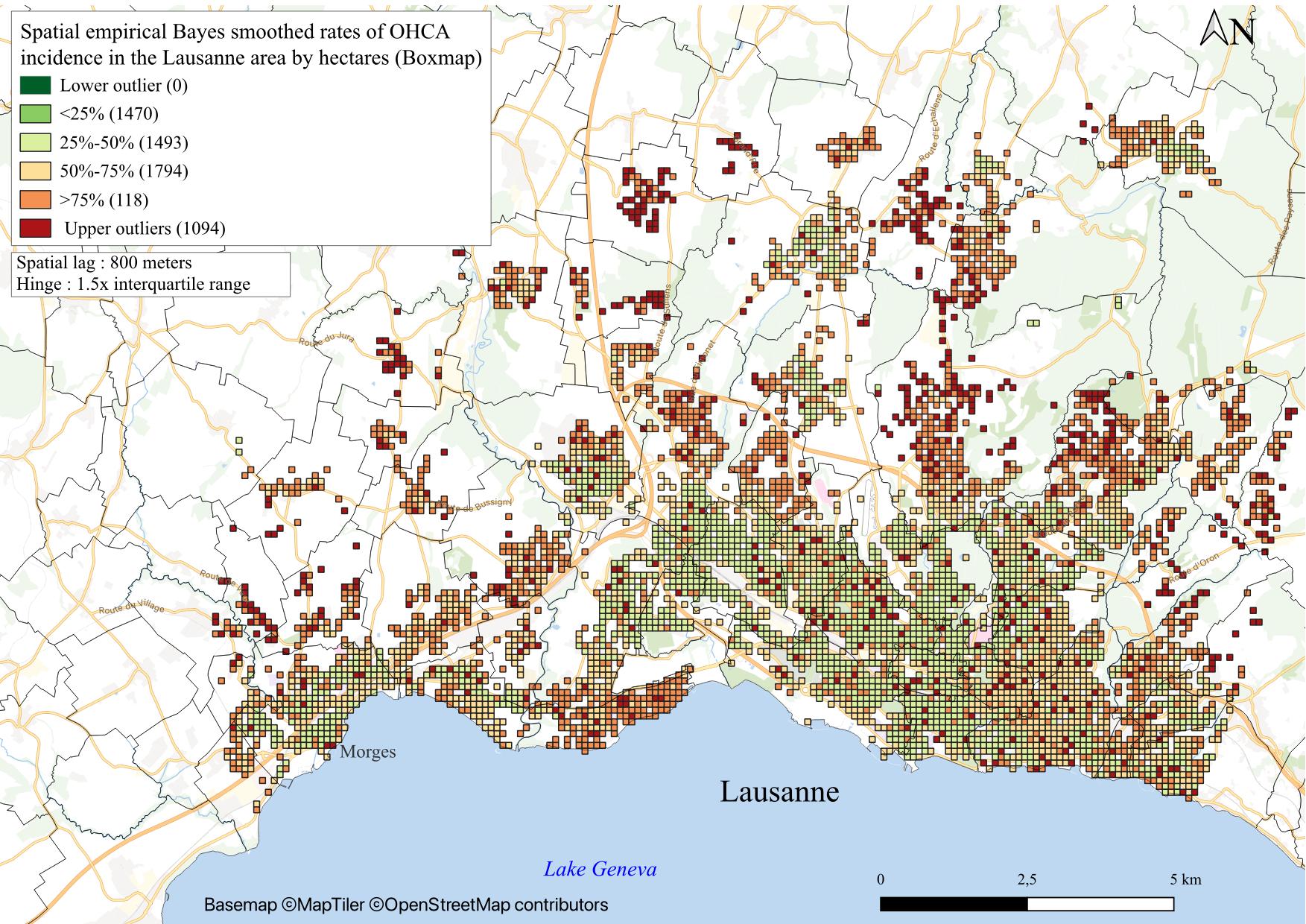


The box map comprises four categories corresponding to the four quartiles, and two supplementary categories corresponding to upper and lower outliers. Green hectares indicate areas with lower OHCA- smoothed rates and red hectares indicate areas with higher OHCA-smoothed rates.

**Fig. B.9.** Empirical Bayes-smoothed out-of-hospital cardiac arrest (OHCA) incidence rates in the canton of Vaud.


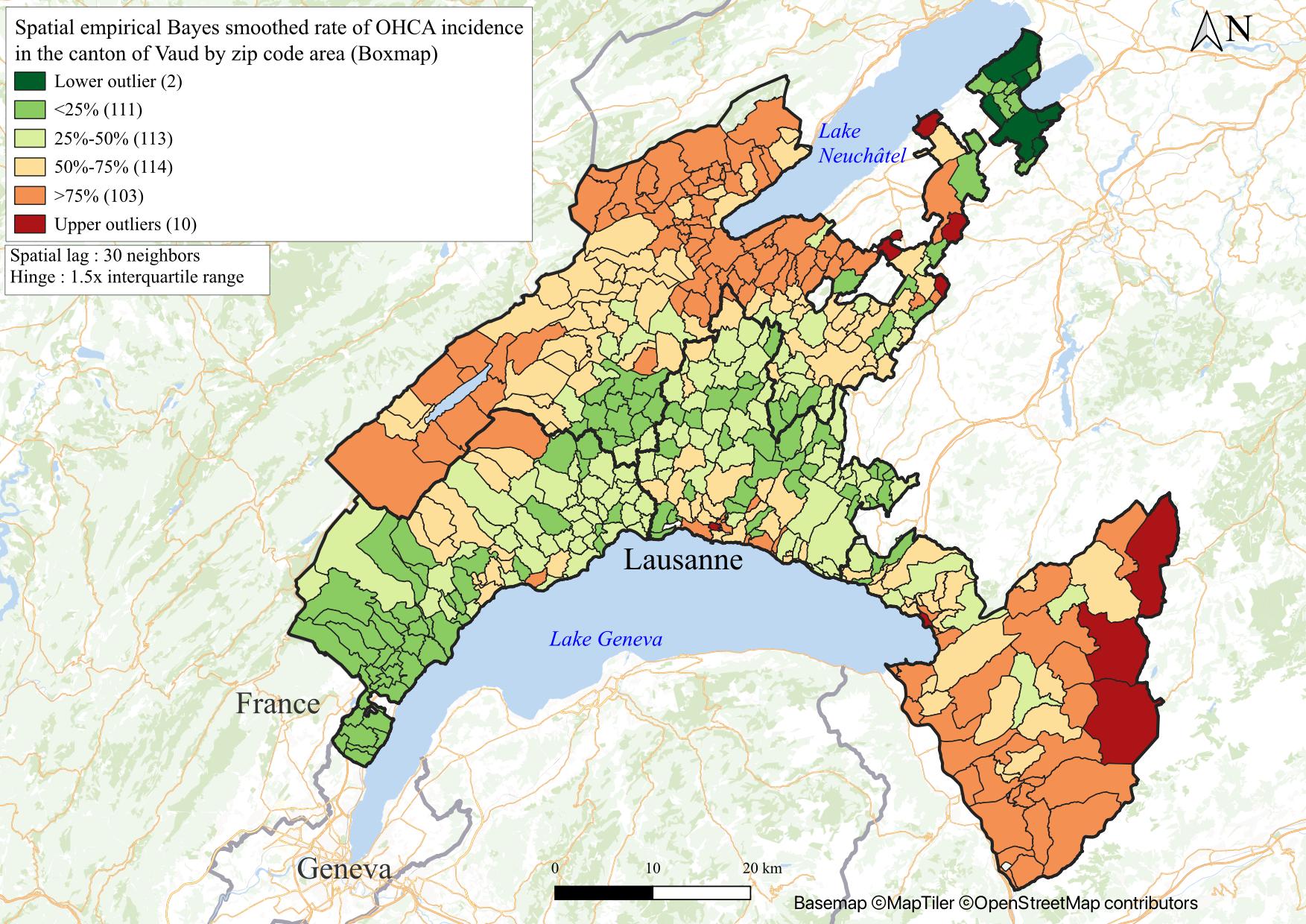


The box map comprises four categories corresponding to the four quartiles, and two supplementary categories corresponding to upper and lower outliers. Green localities indicate areas with lower OHCA- smoothed rates and red localities indicate areas with higher OHCA-smoothed rates.

1. * Statistiques des villes suisses 2023 (2023). Bundesamt für Statistik (BFS). Schweizerischer Städteverband (SSV) / BFS, Bern / Neuchâtel. <https://dam-api.bfs.admin.ch/hub/api/dam/assets/24325655/master>, accessed 09 February 2024.

   *^*^ Detailed explanations of the geographical sectors can be found at: <https://www.lausanne-morges.ch>, accessed 19 June 2024.

   *** Moran P.A.P., Notes on continuous stochastic phenomena Biometrika 1950 17-23 [↑](#footnote-ref-1)
